# On the Hunt for New Toxin Families Produced by a Mediterranean Strain of the Benthic Dinoflagellate *Ostreopsis* cf. *ovata*

**DOI:** 10.3390/toxins14040234

**Published:** 2022-03-23

**Authors:** Eva Ternon, Evgenia Glukhov, Emily Trytten, Rodolphe Lemée, William H. Gerwick

**Affiliations:** 1 Center for Marine Biotechnology and Biomedicine, Scripps Institution of Oceanography, University of California, La Jolla, CA 92093, USA; eglukhov@ucsd.edu (E.G.); etrytten@ucsd.edu (E.T.); wgerwick@health.ucsd.edu (W.H.G.); 2 Laboratoire d’Océanographie de Villefranche (UMR 7093), Sorbonne Université, CNRS, 06230 Villefranche-sur-Mer, France; rodolphe.lemee@imev-mer.fr; 3 Skaggs School of Pharmacy and Pharmaceutical Sciences, University of California, La Jolla, CA 92093, USA

**Keywords:** toxins, *Ostreopsis*, polyhydroxy compounds, cytotoxicity, high resolution mass spectra

## Abstract

*Ostreopsis* cf. *ovata* is a benthic dinoflagellate known to produce palytoxin (PLTX) and its analogues. Recent investigations suggested the production of unknown toxins by a Mediterranean strain. In the present work, two new families of toxins, potentially novel in their structures, were purified from this same Mediterranean strain of *Ostreopsis* cf. *ovata*. The low amount of material isolated only allowed for acquisition of high-resolution mass spectrometry data and the evaluation of their cytotoxicity to human lung cancer cells. Based on their HRMS data, none of these new compounds appear to be close PLTX analogues, although their mass spectra suggest poly-hydroxylated long chain compounds of high molecular weight (1370–2143 Da). The cell cytotoxicity concentrations (CC_50_) of these new purified toxins ranged between 0.68 and 3.12 µg/mL, and this was enhanced when they were tested as mixtures, suggesting synergistic effects of *Ostreopsis* toxins. The two families of compounds were named the liguriatoxins (LGTX) and rivieratoxins (RVTX), with each family containing three members. Additional work on purification is needed to fully characterize the structures of these six new dinoflagellate toxins.

## 1. Introduction

The microalga *Ostreopsis* cf. *ovata* is a benthic dinoflagellate that can causes dermatitis and respiratory syndromes in coastal populations due to the production of active compounds [1,2]. This toxic species, known to be present in the tropics [3], has spread to the Mediterranean Sea [4], and is now dispersing towards higher latitudes, possibly in response to climate change, with frequent occurrence along the Spanish, Portuguese, and French Atlantic coastlines [5,6].

When the first Italian outbreak occurred [1], field samples were screened for known marine biotoxins by liquid chromatography coupled to mass spectrometry (LC-HRMS). One of the detected ions was strongly similar but non-identical to palytoxin (PLTX) [7] and was given the name ovatoxin-a (OVTX-a) [1,8]. The very high molecular weight of OVTX-a (2648.140 Da), its relatively low amount produced by the dinoflagellate cells (<30 pg/cell; [9]) and its physio-chemical properties made its isolation and characterization very challenging. Nevertheless, an exceptional research effort led to the full structural characterization of OVTX-a by nuclear magnetic resonance (NMR) spectroscopy [10].

OVTX-a is not the only PLTX analogue produced by *Ostreopsis* species. Ostreotoxins were found in *O. lenticularis* [11], mascarenotoxins in *O. mascarenensis* [12] and several strains of *O.* cf. *ovata* [3], and ostreocins in *O. siamensis* [13]. Toxins other than PLTX analogues were isolated from a Korean strain of *O.* cf. *ovata* and named ostreols [14,15], but these have never been reported in Mediterranean strains. To date, *Ostreopsis* cf. *ovata* from the North-Western Mediterranean Sea is known to produce up to nine OVTX analogues [8,16,17,18] and low amounts of a putative PLTX [1,8]. The toxin content in *O*. cf. *ovata* varies across different regions; for example, the mascarenotoxins are only produced by strains isolated from the Gulf of Naples [3]. The production of unidentified toxins in strains of *O*. cf. *ovata* collected in the North-Western Mediterranean Sea was indicated by screening chemical fractions of this strain using the *Artemia franciscana* bioassay [19]. Because neither ostreols nor mascarenotoxins were detected in the toxic fractions, new toxins were suspected.

In the present work, a strain of *O.* cf. *ovata* collected from the North-Western Mediterranean Sea (French Riviera, Ligurian Sea) was examined for the presence of new dinoflagellate toxins. Similar challenges faced during OVTX-a characterization were encountered with these new compounds, and NMR spectra could not be acquired due to the small amounts of material available. Therefore, this work focuses on providing mass spectra data for their initial identification and future structure elucidation, together with their biological activity evaluation based on cytotoxicity to the NCI-H460 human lung cancer cell line supplied by ATCC.

## 2. Results

### 2.1. Experimental Section

The extraction and purification procedures yielded only small amounts of pure compounds, ranging between 300 and 700 µg. NMR data acquisitions were attempted but no satisfying spectra were obtained due to the small amount of material combined with the high molecular weights of the compounds. Therefore, we assessed the cytotoxicity of the new compounds, and combined with LC-HRMS traces, compared these data sets to known PLTX analogues.

#### 2.1.1. Bioactivity

**Figure 1:**
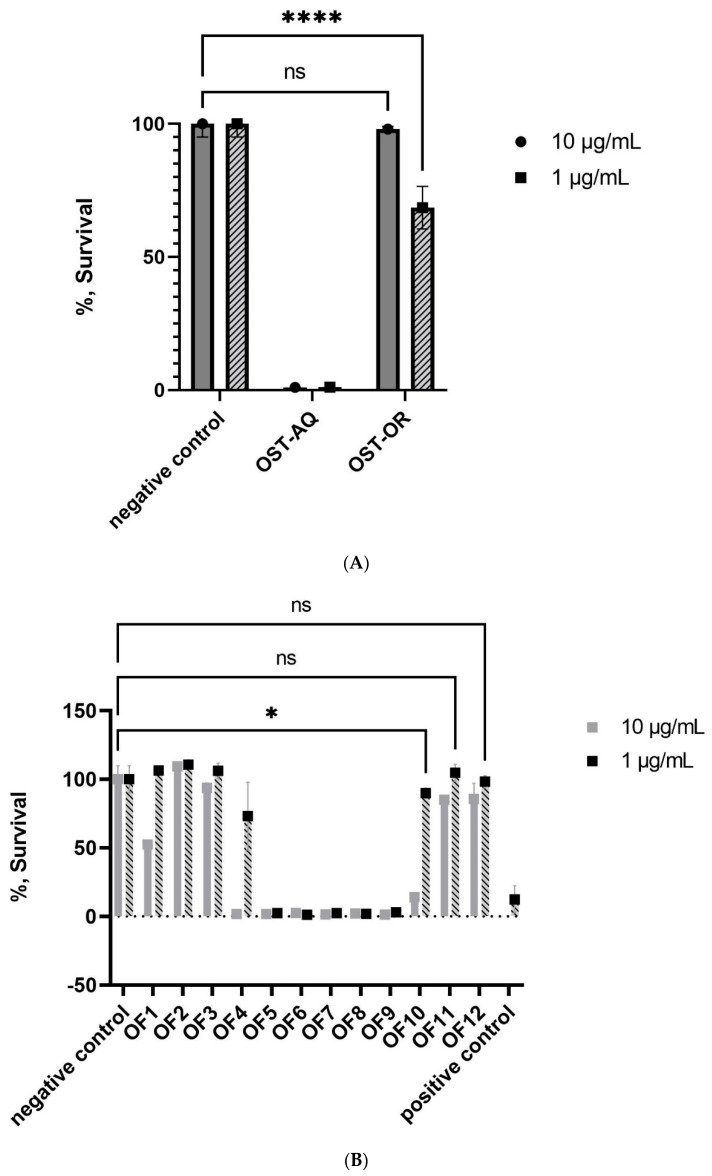

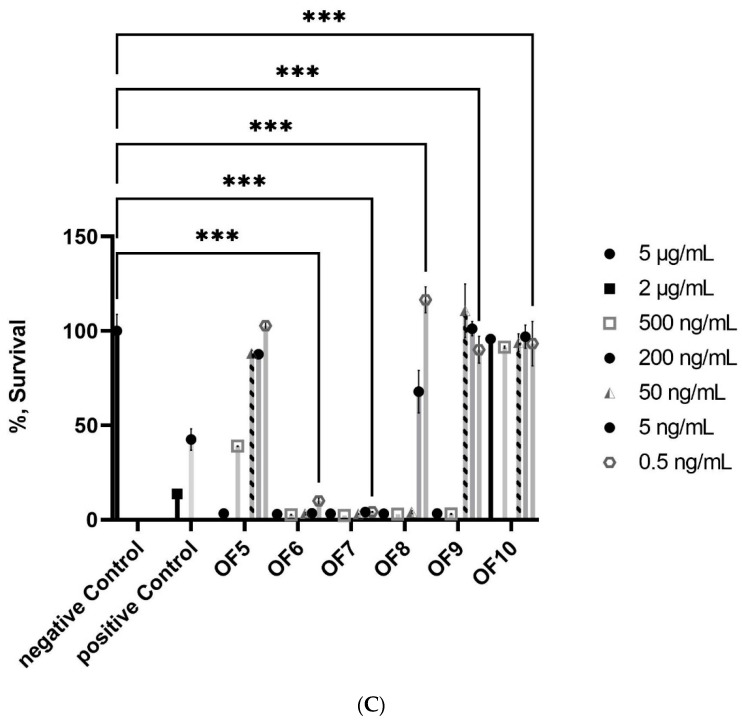
Percent survival of the NCI-H460 cell line as a result of treatment with *O*. cf. *ovata* fractions: (**A**) the aqueous (OST-AQ) and organic (OST-OR) extracts at 1 and 10 µg/mL (**B**) 1 and 10 µg/mL of the fractions OF1 through OF12, and (**C**) 5, 2, 0.5, 0.05, 0.005, and 0.0005 µg/mL of the fractions OF5 through OF10. A concentration of 1% DMSO in RPMI 1640 was chosen to serve as a negative control (set to 100% survival) in all calculations to establish consistency by matching the media DMSO composition of all the samples, while 2 µg/mL doxorubicin in RPMI 1640 served as a positive control. Statistical significance values were calculated using 2-way ANOVA function for panels (**A**) (*p* < 0.0001 ****) and (**B**,**C**) (adjusted *p* = 0.0001 *** and *p* < 0.05 *) (in GraphPad 9.3.1 GraphPad Software Inc., San Diego, CA, USA). No statistical significance was signified using ns.

Both the aqueous and organic extracts obtained from *O.* cf. *ovata* cells exhibited similar cytotoxicity towards NCI-H460 cells at 10 µg/mL, with only 1.2% of the cells that survived (Figure 1A). At 1 µg/mL, only the aqueous fraction showed no cells survival. Up to 67.5% of the NCI-H460 cells survived to the exposition of the organic extract at this concentration.

Based on this activity, the aqueous extract was fractionated by preparative HPLC, yielding twelve fractions. Fractions OF5 to 9 eluting between 9 and 22 min exhibited significant cytotoxicity at 10 and 1 µg/mL (Figure 1B). These five cytotoxic fractions were re-tested at lower concentrations and significant cytotoxicity of fractions OF6, OF7, and OF8 was detected at 50 and 5 ng/mL (Figure 1C). The two most cytotoxic fractions (OF6 and OF7) exhibited an extremely low survival rate of the cells, close to 0%, at 0.5 ng/mL. Only fraction OF7 appeared to contain OVTX, and none of the PLTX analogues or ostreols previously detected in *O*. cf. *ovata* could be found in fraction OF6.

Because most of the compounds comprising fraction OF6 had high molecular weights (>2000 Da) and showed no UV absorbance, a sub-fractionation of this fraction was carried out based on retention time. Eighteen sub-fractions were collected and six of them, OF6-2, -3, -6, -12, -15, and -18 were single compounds (Figure S1) and showed significant cytotoxicity (Table 1). The cell cytotoxicity concentrations (CC_50_) were defined as the fraction or compound concentration dissolved in 1% (*v*/*v*) DMSO, 99% RPMI-1640 that killed 50% of the cells when compared to negative controls (1% (*v*/*v*) DMSO in 99% RPMI-1640 untreated samples).

The OF6 sub-fractions possessed CC_50_ values to NCI-H460 cells between 0.68 and 3.12 µg/mL, compared to 4.46 × 10^−5^ µg/mL for palytoxin (Table 1). The two most cytotoxic fractions were OF6-2 and OF6-6 and had CC_50_ values of 0.68 ± 0.10 and 0.69 ± 0.16 µg/mL, respectively. The least cytotoxic fractions were OF6-12 and OF6-15 with CC_50_ values higher than 1 µg/mL. Assigning a molecular weight for each of these six cytotoxic fractions based on HRMS data, a mixture was further tested for their cytotoxic activity on the same cell line, and the corresponding CC_50_ was considerably reduced (0.14 ± 0.02 µg/mL, Table 1). The concentration–response curves for OF6-2, -3, -6, -12, -15, and -18 are shown in Figure S2, together with a detailed summary of all of the dose response curves (Table S1).

#### 2.1.2. Analytical Chemistry Data

The six compounds that were isolated or partially isolated as sub-fractions from this monoclonal Mediterranean strain of *O*. cf. *ovata* could be segregated into two different groups based on their ionization, isotopic patterns, and molecular weights. A first group of high-molecular weight compounds (around 2000 Da) was present in fractions OF6-2, OF6-3, and OF6-6, and a second group was present in fractions OF6-12, OF6-15, and OF6-18, based on their lower molecular weights < 2000 Da and similar ionization pattern. The two groups were named the liguriatoxins (LGTX) and the rivieratoxins (RVTX), respectively, after the Mediterranean region where the microalgal strain was isolated (i.e., French Riviera in the Ligurian Sea).

**Figure 2:**
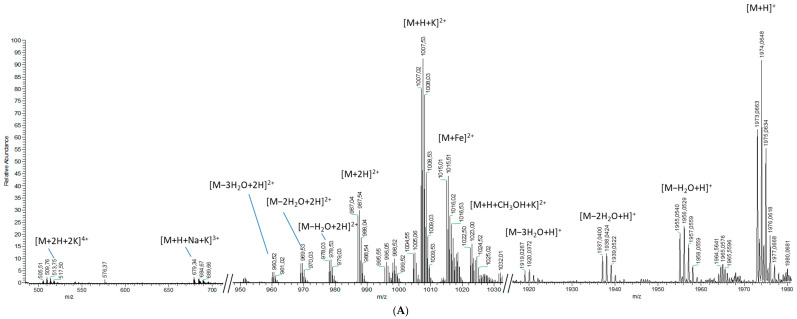

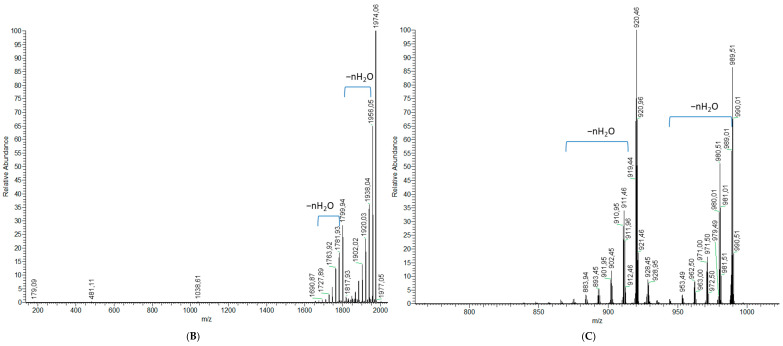
Mass spectra of fractions OF6-2 and -3 (LGTX A) obtained from (**A**) a full scan acquisition, showing the monoisotopic ion and the multi-charged ions (**upper panel**), as well as fragmentation experiments on (**B**) the monoisotopic ion (**lower left panel**), and (**C**) the [M+H+K]^2+^ *m*/*z* 1007.0236 (**lower right panel**).

Fractions OF6-2 and OF6-3 had the same monoisotopic ion at *m*/*z* 1973.0798 (Figure 2A), as well as several [M+H−(H_2_O)_n_]^+^ ions at *m*/*z* 1955.0402, 1937.0432, and 1919.0293 and, therefore, likely contain the same compound, named hereafter as liguriatoxin A (LGTX A). A doubly-charged [M+2H]^2+^ ion at *m*/*z* 987.5399 was detected as well as an intense [M+H+K]^2+^ at *m*/*z* 1007.0236. Five additional doubly-charged ions were detected and resulted from water losses from the [M+2H]^2+^ at *m*/*z* 987.5399. Several adducts were also detected for the doubly-charged ion, such as the [M+Fe]^2+^ at *m*/*z* 1015.0132 and [M+H+CH_3_OH+K]^2+^ at *m*/*z* 1022.5003. Triply and tetra-charged ions were also detected at *m*/*z* 679.0079 and 513.5018, corresponding to the [M+H+Na+K]^3+^ and the [M+2H+2K]^4+^ ions, respectively (Figure 2A).

Fragmentation of the monoisotopic ion led to a major fragment at *m*/*z* 1798.9225 as well as several minor fragments (Figure 2B). Five to six water losses from the monoisotopic ion, as well as from the major fragments, were also detected. Fragmentation of the [M+H+K]^2+^ doubly-charged ion yielded two doubly-charged fragments at *m*/*z* 989.0106 and 919.9597, together with up to five water losses each (Figure 2C). Fragments deriving from the [M+2H]^2+^ species showed only a suite of water losses (data not shown).

**Figure 3:**
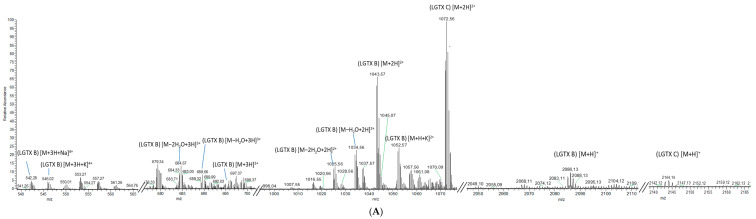

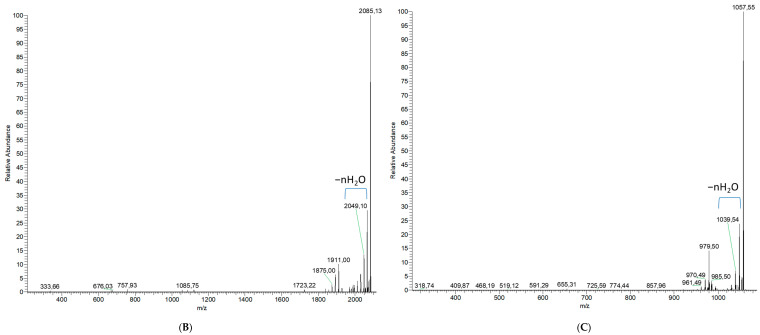
Mass spectra of OF6-6 obtained for (**A**) a full scan acquisition, showing the monoisotopic and the and multi-charged ions of LGTX B and C (**upper panel**), as well as fragmentation experiments on the (**B**) monoisotopic ion of LGTX B (**lower left panel**) and (**C**) the [M+2H]^2+^ ion *m*/*z* 1072.0603 of LGTX C (**lower right panel**).

The analysis of fraction OF6-6 resulted in two [M+H]^+^ peaks at *m*/*z* 2085.1289 and 2143.1438 (Figure 3A), and, therefore, suggested a mixture of two different co-eluting compounds, named liguriatoxin B (LGTX B) and liguriatoxin C (LGTX C), respectively. These two compounds resulted in two doubly-charged [M+2H]^2+^ ions at *m*/*z* 1043.0673 and 1072.0603, respectively (Figure 3A).

LGTX-B showed four water losses in its doubly-charged ion cluster as well as a [M+H+K]^2+^ doubly-charged ion at *m*/*z* 1052.0698. No such adducts or water losses were detected for LGTX C, except for those deriving from its fragment as described below. LGTX B also showed a triply-charged [M+3H]^3+^ ion at *m*/*z* 695.7119 accompanied by two triply-charged ions resulting from water losses at *m*/*z* 689.7093 and 683.7054 (Figure 3A). LGTX C yielded two tetra-charged ions, [M+3H+Na]^4+^ and [M+3H+K]^4+^, at *m*/*z* 542.0254 and 546.0198, respectively (Figure 3A).

The fragmentation of the monoisotopic ion of LGTX B yielded a fragment at *m*/*z* 1910.9886 as well as several minor fragments, including one at *m*/*z* 333.6557 (Figure 3B). Four water losses were also detected from the monoisotopic ion as well as from its main fragment. The [M+2H]^2+^ ion of LGTX B was also fragmented and only yielded a suite of neutral ion water losses (data not shown), and was, therefore, very similar to the fragmentation pattern of the doubly-charged ion [M+2H]^2+^ of LGTX A.

The fragmentation of the [M+2H]^2+^ ion of LGTX C led to one major doubly-charged fragment at *m*/*z* 1057.0552 and several minor fragments at *m*/*z* 978.9940, and 654.8063 (Figure 3C). Several water losses were observed for the higher molecular weight fragments. No common fragments between the two [M+2H]^2+^ ions of LGTX B and C were observed.

**Figure 4:**
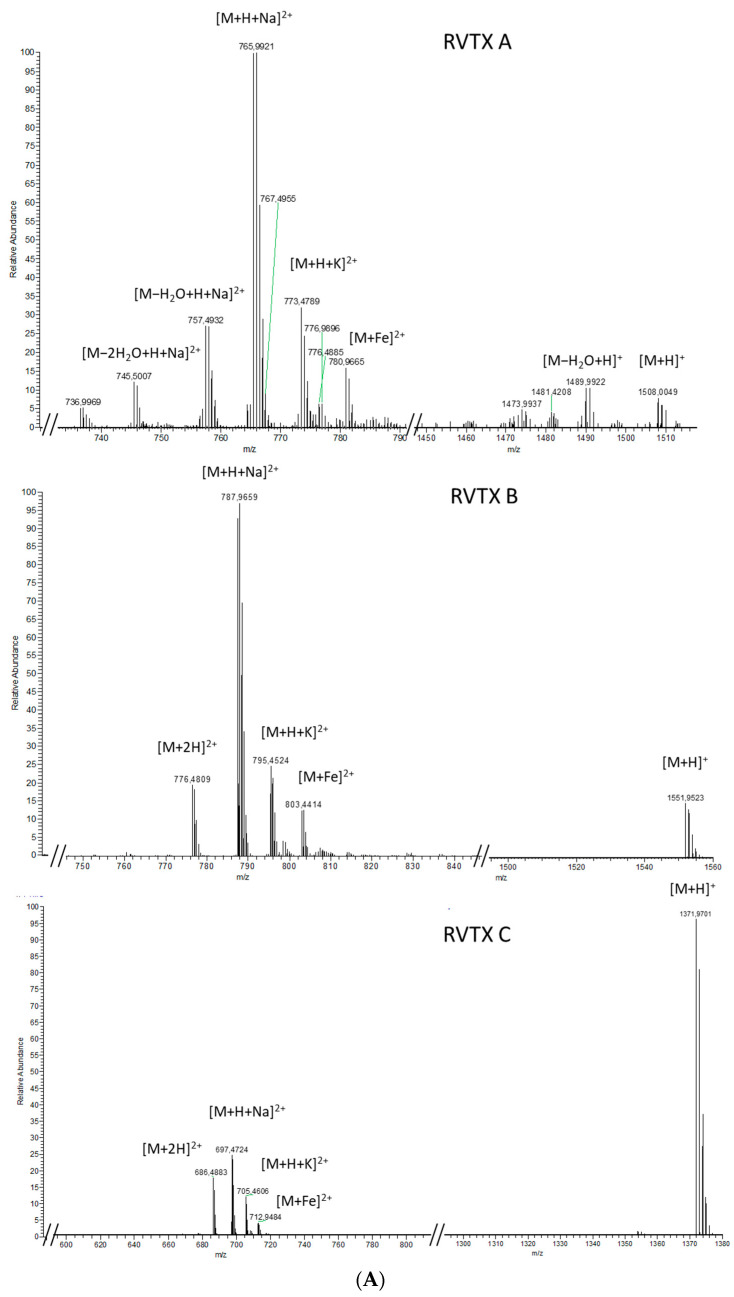

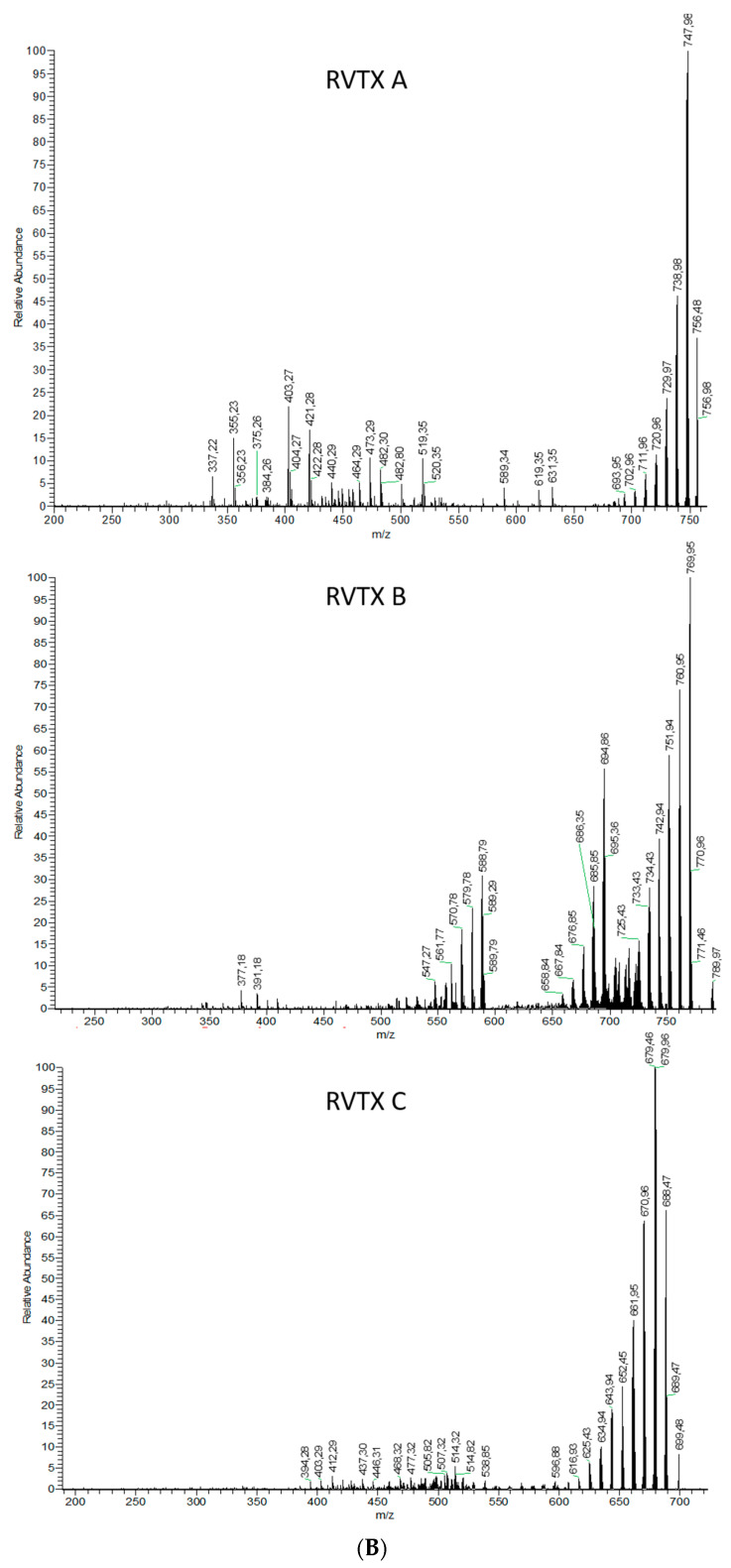
Mass spectra for fractions OF6-12, OF6-15, and OF6-18 (RVTX A, B and C) obtained from (**A**) a full scan acquisition (upper panel) and (**B**) fragmentation of the [M+H+Na]^2+^ ion for each fraction (lower panel).

Whereas the LGTX analogues showed strong similarities in their ionization patterns, they were very different from the three active fractions OF6-12, OF6-15, and OF6-18 (rivieratoxins). These RVTX A, B, and C, exhibited similar patterns to one another with intense doubly-charged ions at *m*/*z* 765.4900, 787.4642, and 697.4731, respectively, corresponding to the [M+H+Na]^2+^ of the [M+H]^+^ ions at *m*/*z* 1508.0032, 1551.9523, and 1371.9681, respectively (Figure 4A).

For the three RVTX compounds, the mass spectra also showed a [M+H+K]^2+^ and a [M+Fe]^2+^, as well as a [M+2H]^2+^ for RVTX B and C. Only RVTX A showed neutral ion water losses from its molecular and doubly-charged ions (Figure 4A), detected at *m*/*z* 1489.9932, 1471.9838, and *m*/*z* 745.500, 736.4957, respectively.

Multiple water losses (up to 10 water molecules) during fragmentation was a shared feature between these three fractions that contained RVTX A–C (Figure 4B). The major fragments resulting from the [M+H+Na]^2+^ of RVTX A, B, and C were *m*/*z* 747.4781, *m*/*z* 769.4520, and *m*/*z* 679.9621, respectively (Figure 4B). At least five ions deriving from these major fragments could be attributed to water losses. Minor fragments between *m*/*z* 200 and *m*/*z* 600 was also observed, but none of these were common between the three RVTX.

**Figure 5:**
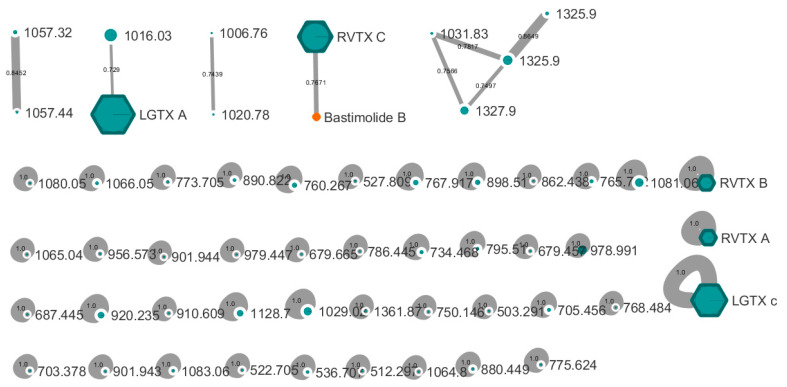
GNPS molecular network obtained from HRMS/MS data of all fractions OF6-2 to OF6-18. All blue nodes were ions detected in at least one fraction, while the orange node corresponds to an ion detected in the library of cyanobacterial natural products. Single nodes mark the absence of common MS^2^ fragments with those of any other compound. The node size corresponds to the precursor ion intensity. The absence of clear fragmentations for LGTX B (only a suite of water losses was observed) precluded its incorporation in the network, whereas LGTX A and C, as well as the RVTX A, B, and C, were all incorporated. Cosine scores on the edges indicate the similarity between two nodes based on their MS^2^ fragments.

A Global Natural Products Social (GNPS) MS^2^-based molecular network was built from all six fractions along with compounds from a standard library of cyanobacteria compounds available on the GNPS platform (Figure 5). However, none of the isolated compounds matched with already known compounds, except RVTX C. This latter compound clustered with the polyhydroxy-macrolide bastimolide B with a cosine score of 0.76.

## 3. Discussion

Two new families of toxins produced by a Mediterranean strain of *Ostreopsis* cf. *ovata,* each containing three analogues, are described in this current study. The three liguriatoxins (LGTX) and three rivieratoxins (RVTX) appear to be unrelated to PLTX analogues based on their HRMS data. Indeed, all PLTX analogues possess a characteristic pattern of fragmentation with two moieties arising from a fragmentation between C-8 and C-9 of the carbon chain [12,13,16,17]. This cleavage yields a characteristic fragment shared by all PLTX analogues and is, therefore, key for their recognition when using mass spectrometry. For example, this diagnostic ion is detected at *m*/*z* 327 for OVTX-a, ostreocins and mascarenotoxins [10,16], while the ions *m*/*z* 371 and *m*/*z* 343 are detected for ovatoxin-b, -c, and -e, respectively [16,20]. These latter ions result from the presence of an additional oxygen or -CH_2_ group in this portion of the molecule. No such characteristic fragments were detected in the mass spectra of the molecules isolated in this current study, a result that is similar to that obtained for the ostreols isolated from Korean strains of *Ostreopsis* cf. *ovata* [14,15]. Prior to the current study, the Korean ostreols are the only family of toxins isolated from *O.* cf. *ovata* that are not PLTX analogues.

None of the liguriatoxins (LGTX) or the rivieratoxins (RVTX) exhibited a UV absorbance between 200 and 600 nm, indicating the absence of any chromophores. In contrast, PLTX analogues typically possess two major chromophores absorbing at 233 and 263 nm [17], corresponding to conjugated unsaturated systems in fragments A and B. Therefore, it can be firmly concluded that neither the LGTX nor the RVTX families share this structural feature.

Even though the new molecules reported herein do not appear to share the same chemical scaffold of the PLTXs, they have in common high molecular weights [10] ranging between 1370 and 2142 Da, multiple water losses (up to 10 molecules) during ionization and fragmentation [8,21], as well as adducts involving a potassium atom [21]. Clearly, all of the isolated compounds are polyhydroxylated compounds, similar to the PLTX analogues, and they may, therefore, share common or similar biosynthetic pathways. In particular, the LGTX metabolites (OF6-2, OF6-3, and OF6-6) possess a high molecular weight (around 2000 Da) as well as multiply-charged ions (two to four charges), a feature shared with the PLTX analogues, suggesting that they may also be polycyclic polyketides although with a shorter carbon chain. The similarity of OF6-18 (RVTX C) to the polyhydroxy macrolide bastimolide B [22], as revealed by the MS^2^-based molecular networking tool (Figure 5), supports the presence of a polyhydroxylated compound with a long carbon chain. Biosynthetically-related metabolites produced by both tropical cyanobacteria and dinoflagellates are poorly documented or understood. However, the cyanobacterium *Trichodesmium* and *Ostreopsis* species both produce PLTX and 42 hydroxy-PLTX [1,23], and, therefore, are expected to possess similar biosynthetic genes for their production. No spectral matches were found for any other fractions in this current study, in particular with any of the OVTXs, thus confirming the novelty of these newly isolated compounds. 

Given their CC_50_ cell cytotoxicity values ranging from 0.68 and 3.12 µg/mL, the new compounds reported here and isolated from *O*. cf. *ovata* can be classified as dinoflagellate toxins. The exposure of NCI-H460 cells (human lung cancer cells) to PLTX yielded a CC_50_ of 0.0446 ± 0.0076 ng/mL, consistent with previous findings on various human cell lines, including breast cancer and keratinocyte cells (EC_50_ = 0.00197 to 0.19 ng/mL; [24,25,26]). PLTX analogues are known to be less toxic than PLTX itself, with EC_50_ values (Half maximal Effective Concentration) ranging between 1.19 to 8.72 ng/mL for ostreocin D [25,26], and 0.41 ng/mL for OVTX-a [26]. Thus, the cytotoxic potency of the novel toxins (680–3120 ng/mL) described herein is lower compared to PLTX analogues. Although suspected to be the same compound based on their HRMS data, fractions OF6-2 and OF6-3 (LGTX A) did not exhibit the same biological activity, and this may be attributed to impurities present in fraction OF6-3 (Figure S1). Similarly, fraction OF6-6 resulted in a mixture of two compounds (LGTX B and C) and was associated with the highest cytotoxic effects (0.69 ± 16 µg/mL) found among these materials. As a mixture, the effects of the LGTX B and C may be enhanced, as observed for cyanobacterial lipopeptides [27], and their separation is necessary to better estimate their individual activities. Synergistic effects of polyhydroxylated dinoflagellate toxins have been poorly documented to date [26]. Interestingly, in the current study a significant increase in the CC_50_ (0.14 ± 0.02 µg/mL) was observed when testing for synergism between the isolated LGTX’s and the RVTX’s on human lung cancer cells. Synergistic effects between the new set of toxins and minor metabolites present in the initial fraction OF6 may also explain its exceptionally high potency (90% of mortality at 0.05 ng/mL) compared to the completely isolated compounds. Nevertheless, at this stage we cannot exclude the possibility that fraction OF6 contains a highly cytotoxic minor compound that could have been isolated as a separate and distinct species. This contrasts with the OVTXs, which did not show enhanced cytotoxic effects when tested as a mixture (4.8 against 6.6 ng/mL using an ELISA assay [26]). A mixture of the new set of toxins and the OVTX’s would be expected to occur in natural conditions and in various environmental matrices (particles, water, aerosols); such combined or synergistic toxicities should be better characterized in future studies.

Pelin et al. (2016) attempted to identify structural features that were responsible for the high toxicity of PLTX analogues and suggested a pivotal role of the hydroxy group at position C44. Even though no such chemical structure could be firmly identified from the HRMS data in this current study, these new toxins also contain a large number of hydroxy groups, as suggested by the multiple water losses during MS ionization, and these could be responsible in part for the observed cytotoxicity. These results lay the foundation for future work on the structural elucidation of the liguriatoxins (LGTX) and the rivieratoxins (RVTX). Additionally, the detection of this set of new toxins may be included in monitoring efforts in the Mediterranean Sea, since they present significant toxicity on human cell lines, especially when present as a mixture.

## 4. Methods

### 4.1. Culture

A monoclonal strain of *O*. cf. *ovata* obtained from the MCCV (Mediterranean Culture Collection of Villefranche, MCCV54, isolated from the Villefranche Bay, France) was grown in 120 L of L1 medium prepared with autoclaved, aged, and filtered natural seawater (collected from the Scripps Institution of Oceanography pier, La Jolla, CA, USA) and adjusted to a salinity of 38 ppt with NaCl. The cultures were maintained for ten days at 23 °C under a 14:10 light/dark cycle with a light intensity of 250 µmol/m^2^/s, at which time the cells were harvested by centrifugation at 1800 rpm for 12 min and the supernatant was discarded. Ten days of growth was previously identified as the peak for toxin production in this strain [9,28].

### 4.2. Compound Purification

The cell pellets were freeze dried and weighed. A total of 3.5 g of dried cells were extracted with a mixture of H_2_O/MeOH (20:80) by sonication while cooling in an ice bath for 10 min. The extract was centrifuged at 3500 rpm for 10 min and the supernatant was transferred to a round bottom flask for in vacuo evaporation. Two additional extractions were performed, and all supernatants were combined. The organic phase of the extract was evaporated at 38 °C with a rotavapor and the remaining aqueous phase was further extracted three times with diethyl ether using a separatory funnel. The diethyl ether fractions were combined, evaporated to dryness, and stored at −20 °C. Aliquots of both the aqueous and the diethyl ether fractions were assessed for their cytotoxicity.

The aqueous fraction was freeze dried and resuspended in 2 mL of MeOH/H_2_O (90:10). Centrifugation of these 2 milliliter samples at 4000 g for 7 min pelleted most of the salts that were present. The supernatant was transferred to a new vial and purified by prep-HPLC on a Luna C18 column (Kinetex, 250 × 21.2 mm, 5 µ). A gradient of H_2_O/ACN was set from 10% to 100% of ACN over 34 min at a flow rate of 21 mL/min. Detection was performed at 210 nm, and 8 injections of 2 mL each were carried out. Final purification of the toxins was achieved by HPLC on a Dionex UltiMate 3000 (Thermo Scientific, Waltham, MA, USA) system using an AQUA C18 column (Phenomenex, 250 × 4.6 mm, 5 µ), an RS diode array detector, and an automated fraction collector. The toxins were eluted using isocratic conditions (H_2_O/ACN, 65:35, *v*/*v*) over 30 min at a flow rate of 0.6 mL/min. Optimal detection of the OVTX was achieved using wavelengths set at 210, 254, 263, and 280 nm. The isolation of the new compounds was based on their retention time, preliminarily assessed by LC-MS using the same chromatographic conditions on a Thermo Finnigan Surveyor Autosampler-Plus/LC-Pump-Plus/PDA-Plus system coupled to a Thermo Finnigan LCQ Advantage Max mass spectrometer (Thermo Scientific, Waltham, MA, USA), monitoring 200–600 nm and *m*/*z* 200–2000 in positive ion mode.

### 4.3. LC-HRMS/MS Analysis

Mass spectrometry data from the different fractions were acquired by UHPLC-HRMS, using a Thermo Scientific Vanquish system (Agilent Technologies, Santa Clara, CA, USA). Separation was achieved on a UPLC C18 column (Kinetex^®^ 2.6 µm, 150 × 2.1 mm, Phenomenex, Torrance, CA, USA) maintained at 40 °C. Eluent A was water and Eluent B was acetonitrile (ACN, LC-MS grade, Sigma, St Louis, MI, USA), both eluents containing 0.1% of acetic acid. A gradient elution from 20 to 95% of B over 10 min was applied at a flow rate of 400 µL/min and the injection volume was 5 µL. Mass spectral data were acquired using an Orbitrap Elite MS mass spectrometer (Agilent Technologies, Santa Clara, CA, USA) equipped with an electrospray ionization in the positive mode. Full scan spectra were acquired in the range 200–2000 Da, in the CID mode with a FTMS Analyzer set at a resolution of 120,000. Collision energies (CE) of 40 eV were applied to obtain the MS/MS spectra. The capillary voltage of the MS spectrometer was set at 3500 V (positive mode), and the nebulizing parameters were set as follows: nebulizing gas (N_2_) pressure at 0.5 bar, drying gas (N_2_) flow at 11 L/min, the drying temperature at 300 °C, and the Vaporizer/Sheath Gas Temp, 300 °C.

The raw data were converted to MZxml files using the open-source MSconvert (Proteowizard^®^) and were submitted to the on-line GNPS platform to evaluate for structural similarities with known compounds listed in the GNPS libraries [29].

### 4.4. Cytotoxicity Assays

Evaluation of compounds for cytotoxicity was performed in vitro using the commercial cell line of NCI-H460 human large cell lung carcinoma cell line purchased from American Type Culture Collection (ATCC) in December 2019 and the MTT cell viability method [30]. Cell monolayers were grown to near confluence in 75 cm^2^ cell culture flasks in RPMI 1640 medium (Corning cat. 10040CV, Corning, NY, USA) with 10% qualified FBS (Gibco cat. 10437028, Waltham, MA, USA), 1 mM sodium pyruvate (Corning cat 25-00-CL, Corning, NY, USA), 0.15% sodium bicarbonate (Corning cat 25080094, Corning, NY, USA) as well as Penicillin-Streptomycin 100 X solution (HyClone cat. SV30010, Logan, UT, USA). Detached cells were seeded at 3.33 × 10^4^ cells/mL in 96-well flat bottom wells with low evaporation lid plates and 180 µL/well. Following 24 h of incubation at 37 °C, cells were exposed to the samples dissolved in cell media with 1% DMSO but without fetal bovine serum (FBS). Initially, dried samples (either mixtures or isolated compounds) were resuspended in DMSO, then diluted with RPMI 1640 medium to reach 10 times of the desired final concentrations and were further added at 20 µL/well. Each sample’s concentration was tested in duplicate in three independent experiments. Plates were incubated for an additional 48 h at which time the medium was aspirated, and cells stained with 1 mg/mL of MTT (thiazolyl blue tetrazolium bromide 98%; Sigma-Aldrich, St Louis, MI, USA) for 25 min at 37 °C. Their absorbance was analyzed at 570 and 630 nm in a 96-well SpectraMax M3 microplate reader (Molecular Devices LLC, San Jose, CA, USA) after 55 s of orbital shaking. Cell viability was calculated against a negative control of 1% DMSO in RPMI 1640 without FBS (set as 100% cell viability or 0% death). Doxorubicin in RPMI 1640 without FBS was used as the positive control. Pure compounds were initially dissolved in DMSO and brought to a starting concentration, and then nine more working solutions were made through serial dilution using a factor of 0.3164 with RPMI 1640 media. DMSO concentration was maintained at 1% in all cell exposed mixtures. CC_50_ values were obtained from concentration–response curves from duplicate tests of average cell viability values versus drug concentrations employing GraphPad Prism 9.3.1 (GraphPad Software Inc., San Diego, USA)and using a non-linear regression fit [log (inhibitor) vs. response-Variable slope (four parameters)] fitting model. Final CC_50_ average values as well as standard deviations were calculated from the three independent experiments. For the most potent compounds, the initial screening concentrations were reduced in additional experiments in order to construct full dose response curves. Authentic PLTX was purchased from Wako chemicals (GmbH, Neuss, Germany).

## Figures and Tables

**Table 1:** CC_50_ mean values and standard deviations obtained for the isolated compounds, their mixture, and PLTX. Values are expressed in weight per volume (µg/mL), each value was a result of three concentration–response curves.

Compound	CC_50_ (µg/mL)
OF6-2	0.68 ± 0.1
OF6-3	1.40 ± 0.08
OF6-6	0.69 ± 0.16
OF6-12	3.12 ± 0.13
OF6-15	1.28 ± 0.24
OF6-18	0.76 ± 0.17
OF6-mix	0.14 ± 0.02
PLTX	4.46 × 10^−5^ ± 0.76 × 10^−5^
Doxorubicin	0.18 ± 0.03

## Data Availability

The LC-HRMS data are available on the MassIVE repository ID=38f9fc0a66e643b593bc7674e3f71c52.

## Supplementary Materials

The following supporting information can be downloaded at https://www.mdpi.com/article/10.3390/toxins14040234/s1, Table S1: Overall summary of NCI-H460 cell line concentration–response curves including CC_50_ and R^2^ values (≥0.89) for each curve. Cell cytotoxicity values are expressed in µg/mL; Figure S1: Low-resolution mass spectra for the LGTX (top panel) and the RVTX (bottom panel) acquired in the full scan positive mode; Figure S2: Concentration–response curves for all purified fractions of OF6-2 to 6, LGTX (top panel) and the OF6-12 to 18, RVTX (second panel), as well as their mixture (OF6-MIX), PLTX, and doxorubicin (positive control) (bottom panel), concentrations expressed as weights/volume (µg/mL). Curves were created using log (inhibitor) vs. response-Variable slope (four parameters) function prepared in GraphPad 9.3.1.
