# Supplementary material for: On the Hunt for New Toxin Families Produced by a Mediterranean Strain of the Benthic Dinoflagellate Ostreopsis cf. ovata
Source: Toxins (Basel). 2022 Mar 23;14(4):234. doi: 10.3390/toxins14040234 (PMC9030729; doi:10.3390/toxins14040234)
Supplement: Supplementary file 1 [file toxins-14-00234-s001.zip › toxins-1622573-supplementary.pdf]

toxins MDPI

Article

On the Hunt for New Toxin Families Produced by a Mediterranean Strain of the Benthic Dinoflagellate *Ostreopsis* cf. *ovata*

Eva Ternon, Evgenia Glukhov, Emily Trytten, Rodolphe Lemée and William Gerwick

Table S1. Overall summary of NCI-H460 cell line concentration-response curves including CC_{50} and R^2 values (≥ 0.89) for each curve. Cell cytotoxicity values are expressed in µg/mL.

COMPOUND ID	A	B	C	AVG, µg/mL	error, µg/mL	% ERROR	R2 A	R2 B	R2 C
**OF6-2**	5.65E-01	7.20E-01	7.46E-01	**0.68**	**0.10**	**10**	0.9711	0.9748	0.986
**OF6-3**	1.41E+00	1.49E+00	1.32E+00	**1.40**	**0.08**	**8**	0.9144	0.8721	0.96
**OF6-6**	5.24E-01	8.38E-01	7.07E-01	**0.69**	**0.16**	**16**	0.9831	0.9686	0.9807
**OF6-12**	3.06E+00	3.28E+00	3.04E+00	**3.12**	**0.13**	**13**	0.9468	0.981	0.9522
**OF6-15**	1.12E+00	1.56E+00	1.16E+00	**1.28**	**0.24**	**24**	0.8853	0.9812	0.9449
**OF6-18**	9.56E-01	6.80E-01	6.59E-01	**0.76**	**0.17**	**17**	0.9462	0.9801	0.9748
**PLTX**	4.02E-05	5.34E-05	4.04E-05	**4.5E-05**	**7.6E-06**	**0**	0.9907	0.9864	0.9665
**OF6-MIX**	1.58E-01	1.36E-01	1.26E-01	**0.14**	**0.02**	**2**	0.9884	0.9908	0.9543
**DOX**	1.49E-01	2.02E-01	1.87E-01	**0.18**	**0.03**	**3**	0.9866	0.9728	0.9457

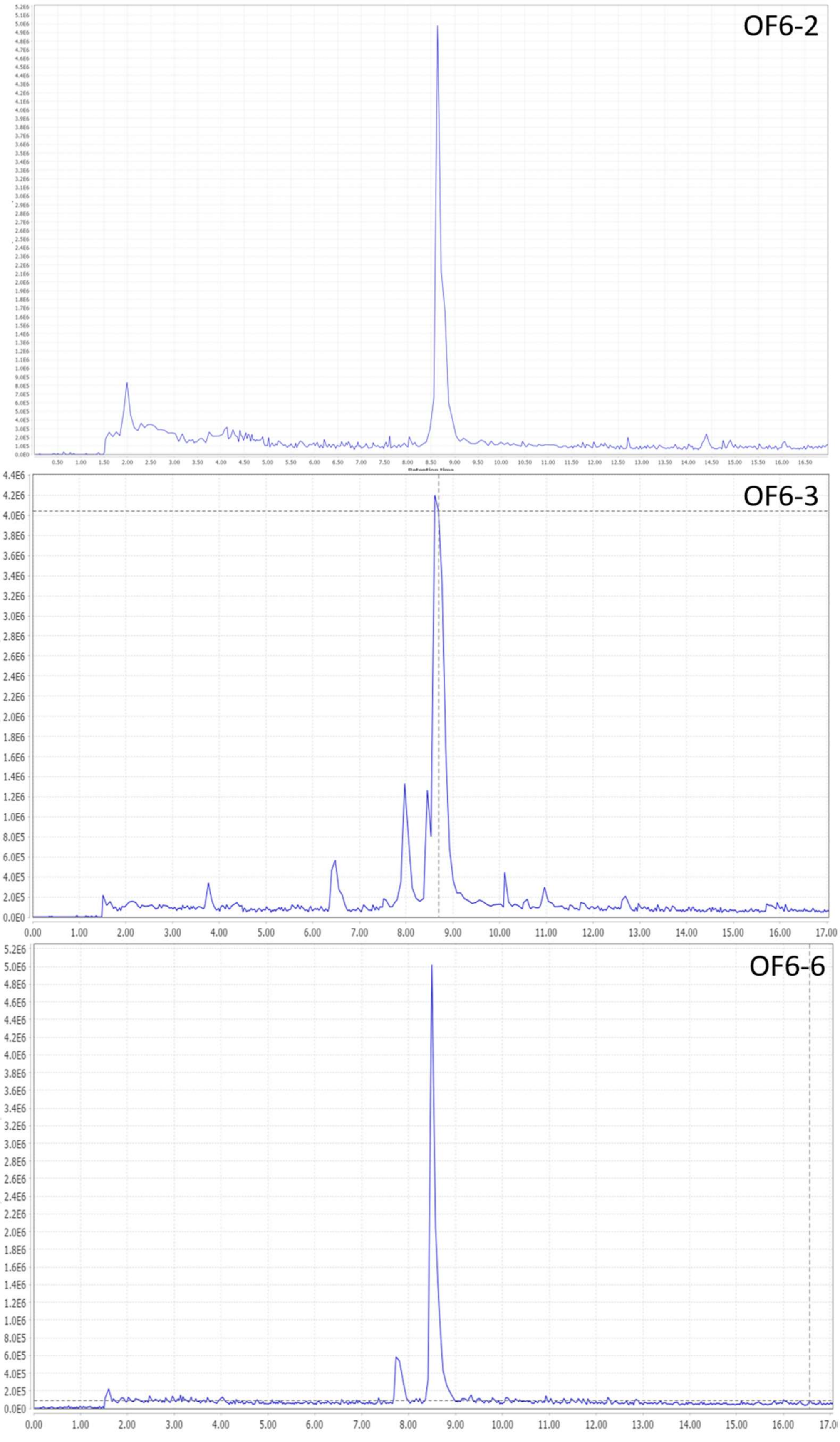

(A)

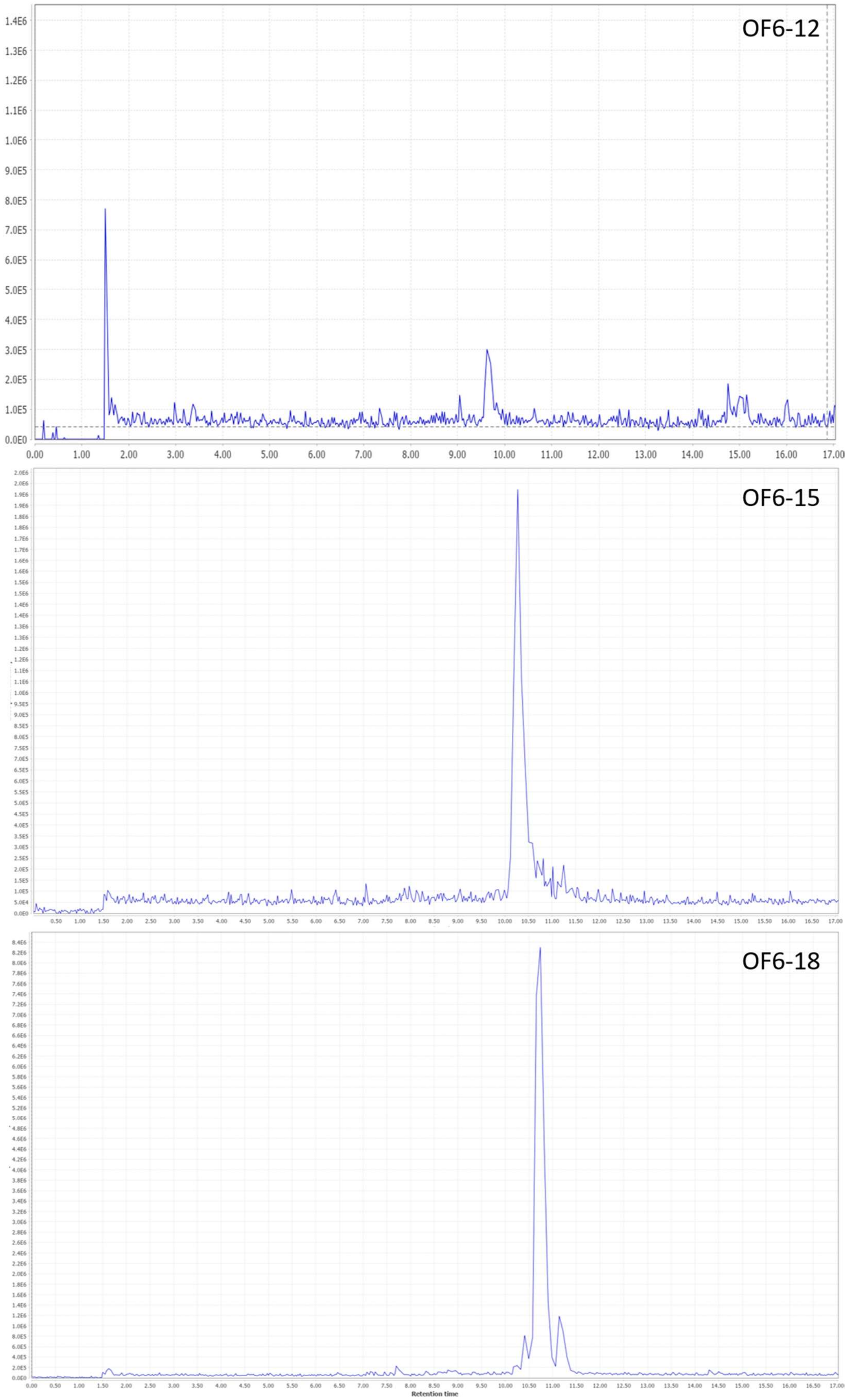

(B)

Figure S1. (A) Low-resolution mass spectra for the LGTX (top panel) and (**B**) the RVTX (bottom panel) acquired in the full scan positive mode.

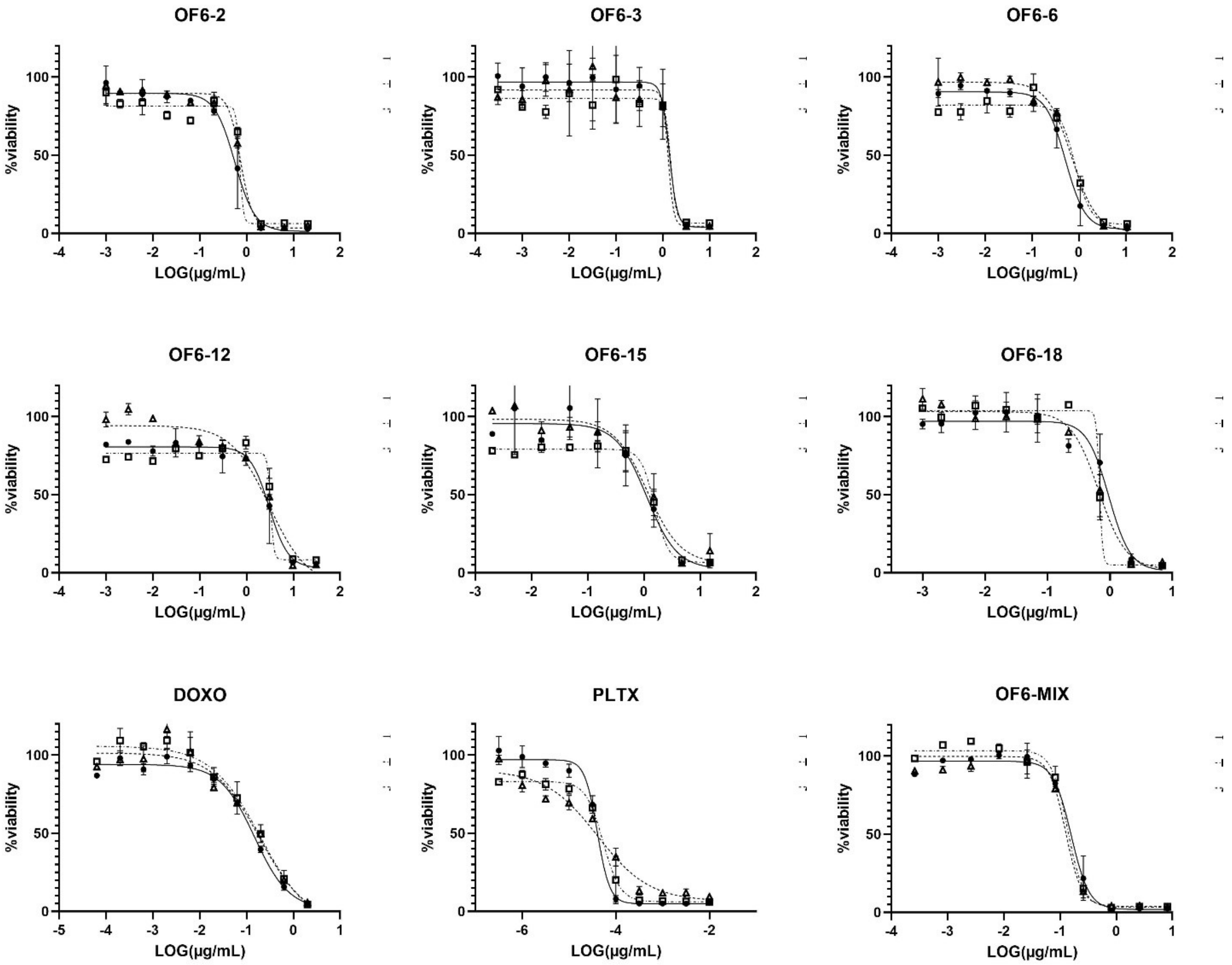

Figure S2. Concentration-response curves for all purified fractions of OF6-2 to 6 , LGTX (top panel) and the OF12-18, RVTX (second panel), as well as their mixture (OF6-MIX), PLTX and doxorubicin (positive control) (bottom panel), concentrations expressed as weights/volume (µg/mL). Curves were created using log(inhibitor) vs. response -- Variable slope (four parameters) function prepared in GraphPad 9.3.1.
